# IL-1β reactivity and the development of severe fatigue after military deployment: a longitudinal study

**DOI:** 10.1186/1742-2094-9-205

**Published:** 2012-08-21

**Authors:** Mirjam van Zuiden, Annemieke Kavelaars, Karima Amarouchi, Mirjam Maas, Eric Vermetten, Elbert Geuze, Cobi J Heijnen

**Affiliations:** 1 Laboratory of Neuroimmunology and Developmental Origins of Disease (NIDOD), University Medical Center Utrecht, KC.03.068.0, P.O. Box 85090, 3508 AB, Utrecht, the Netherlands; 2 Research Centre - Military Mental Health, Ministry of Defence, Lundlaan 1, 3584 EZ, Utrecht, the Netherlands; 3 Department of Psychiatry, Academic Medical Center, University of Amsterdam, Meibergdreef 5, 1105 AZ, Amsterdam, the Netherlands; 4 Integrative Immunology and Behavior Program, University of Illinois Urbana Champaign, 61801, Urbana, IL, USA; 5 Department of Psychiatry, Rudolf Magnus Institute of Neuroscience, University Medical Center Utrecht, Heidelberglaan 100, 3584 CX, Utrecht, the Netherlands

**Keywords:** Fatigue, Stress, Inflammation, Cytokine, Interleukin-1, Receptor, Reactivity, Military, LPS, Interleukin-8

## Abstract

**Background:**

It has been suggested that pro-inflammatory cytokine signaling to the brain may contribute to severe fatigue. We propose that not only the level of circulating cytokines, but also increased reactivity of target cells to cytokines contributes to the effect of cytokines on behavior. Based on this concept, we assessed the reactivity of peripheral blood cells to IL-1β *in vitro* as a novel approach to investigate whether severe fatigue is associated with increased pro-inflammatory signaling.

**Methods:**

We included 504 soldiers before deployment to a combat-zone. We examined fatigue severity and the response to *in vitro* stimulation with IL-1β prior to deployment (T0), and 1 (T1) and 6 months (T2) after deployment. IL-8 production was used as read-out. As a control we determined LPS-induced IL-8 production. The presence of severe fatigue was assessed with the Checklist Individual Strength (CIS-20R). Differences in dose–response and the longitudinal course of IL-1β and LPS-induced IL-8 production and fatigue severity were investigated using repeated measures ANOVA.

**Results:**

At T2, the group who had developed severe fatigue (*n* = 65) had significantly higher IL-1β-induced IL-8 production than the non-fatigued group (*n* = 439). This group difference was not present at T0, but developed over time. Longitudinal analysis revealed that in the non-fatigued group, IL-1β-induced IL-8 production decreased over time, while IL-1β-induced IL-8 production in the fatigued group had not decreased. To determine whether the observed group difference was specific for IL-1β reactivity, we also analyzed longitudinal LPS-induced IL-8 production. We did not observe a group difference in LPS-induced IL-8 production.

**Conclusions:**

Collectively, our findings indicate that severe fatigue is associated with a higher reactivity to IL-1β. We propose that assessment of the reactivity of the immune system to IL-1β may represent a promising novel method to investigate the association between behavioral abnormalities and pro-inflammatory cytokine signaling.

## Background

The experience of prolonged severe fatigue after return from military deployment is a common phenomenon. The prevalence of severe fatigue in Dutch military personnel 1 to 4 years after return from deployment to Cambodia, Rwanda, and Bosnia has been estimated to be 7.6 to 12.4 times higher than in non-deployed military personnel [1]. In addition, the prevalence of chronic fatigue syndrome (CFS)-like symptoms in US military personnel 5 years after return from deployment to the Gulf Region was 6.8 to 9.1 times higher compared to non-deployed military personnel [2].

It has been suggested that the development of severe fatigue may result from behavioral consequences associated with increased pro-inflammatory signaling [3-7]. An increase in pro-inflammatory signaling may result from increased levels of circulating pro-inflammatory cytokines. Consistent with this notion, increased levels of circulating pro-inflammatory cytokines have repeatedly been observed in individuals with severe fatigue or CFS compared to non-fatigued individuals [8-13]. However, not all results of studies on cytokines levels in fatigue are consistent with increased levels of circulating pro-inflammatory cytokines: decreased or unaltered levels of pro-inflammatory serum cytokine levels also have been described in severely fatigued individuals compared to non-fatigued individuals [3-7].

The response of the body to an inflammatory mediator or other regulatory mediators does not only depend on the circulating levels of the specific mediator at a given moment, but also depends on the sensitivity or reactivity of the target system to regulation by the specific mediator. This reactivity of the target cells is determined at the level of the receptor, by receptor number, ligand binding affinity, and coupling of the receptor to intracellular signaling pathways. In addition, intracellular processes downstream of the receptor determine the reactivity of a cell to regulation by specific mediators [14]. Thus, an increase in pro-inflammatory signaling may also result from increased reactivity of target cells to pro-inflammatory cytokines.

Based on this concept, we assessed IL-1β-induced cytokine production by peripheral blood cells *in vitro* to determine whether severe fatigue is associated with altered reactivity of immune cells to pro-inflammatory cytokines. We selected IL-1β because of the existing evidence for a pivotal role of IL-1β signaling in the behavioral consequences of inflammation. For example, systemic or central administration of IL-1β triggers the development of sickness behavior in rodents [15]. Moreover, the development of peripheral inflammation-induced sickness behavior in rodents can be completely prevented when IL-1 action is blocked [16]. Furthermore, the fatigue symptoms of patients with the chronic inflammatory disease rheumatoid arthritis were significantly reduced after administration of an IL-1-receptor antagonist [17]. In peripheral blood mononuclear cells, IL-1β induces the production of pro-inflammatory cytokines and chemokines, including IL-8 [18]. Therefore altered IL-8 production by peripheral blood mononuclear cells in response to exposure of these cells to IL-1β is an indicator of altered reactivity of IL-1 receptors and/or downstream signaling pathways.

We assessed whether soldiers with and without severe fatigue 6 months after return from deployment to a combat-zone differed in IL-1β-induced IL-8 production by peripheral blood cells, as assessed *in vitro* at 6 months (T2) after return from deployment. We also investigated the longitudinal course of IL-1β-induced IL-8 production in samples obtained prior to (T0), 1 month (T1), and 6 months after deployment (T2).

Our results show that soldiers with severe fatigue showed a higher reactivity to IL-1β at 6 months after return from military deployment than the non-fatigued group. This group difference had developed in response to the deployment. These results indicate that assessment of the reactivity of the immune system to IL-1β may be a promising novel method to study the association between behavioral abnormalities and pro-inflammatory cytokine signaling.

## Methods

### Ethics statement

This study was carried out in compliance with the Declaration of Helsinki. The study was approved by the Institutional Review Board of the University Medical Center Utrecht, the Netherlands. Written informed consent was obtained after a written and verbal description of the study.

### General procedure

This study is part of a prospective cohort study on biological and psychological aspects of the development of deployment-related disorders in the Dutch Armed Forces [19-24]. Military personnel of the Dutch Armed Forces assigned to a 4-month deployment were included on a voluntary basis. Their duties during deployment included combat patrols, clearing or searching buildings, participation in de-mining operations, and transportation across enemy territory. Typical combat-zone stressors included enemy fire, armed combat, and combat casualties. We included participants deployed from 2006 to 2009. Participants were assessed 1 to 2 months prior to deployment (T0), and approximately 1 (T1) and 6 months (T2) after their return from deployment. During each assessment, participants filled out questionnaires. In addition, a heparinized blood sample was drawn between 08:00 and 11:30. Heparinized blood was kept at room temperature.

### Participants

A total of 721 participants completed questionnaires and blood sampling for measurement of IL-1β sensitivity before deployment (T0). Since we were interested in the development of severe fatigue in response to deployment, we excluded 32 (4.4%) participants who already reported severe fatigue before deployment, resulting in 689 participants at T0. Twelve participants (1.7%) were not available for follow-up (non-deployed (*n* = 10); deceased during deployment (*n* = 2)). Of the eligible 677 participants after deployment, 504 participants (74.4%) completed the assessments at T1 and T2.

Participants were divided into groups based on their level of fatigue at T2, assessed with the Checklist Individual Strength (CIS-20R). The used cutoff for the total score on the CIS-20R was ≥81 [19]. This cutoff corresponds to the 95th percentile of scores before deployment within a population of 862 Dutch military personnel (mean (SD): 45.87 (17.69)).

A total of 65 participants (12.9%) reported severe fatigue at T2 and were therefore included in the fatigued group. The remaining 439 participants (87.1%) were included in the non-fatigued group.

Compared to eligible individuals who did complete the assessments after deployment, dropouts were younger during deployment (mean (SD): dropouts: 25.89 (6.08); completers: 28.57 (8.98), t_(673)_: -3.64, *P* < 0.001). As a result they had been deployed less often (mean (SD): dropouts: 0.64 (0.93); completers: 0.91 (1.22), t_(650)_: -2.33, *P* < 0.05) and were lower ranked $\left({\chi^{2}}_{\left(3\right)}:13.91,\text{p}<.01\right)$. There was no significant difference in educational level between completers and dropouts $\left({\chi^{2}}_{\left(2\right)}:4.50,\text{p}=.105\right)$. In addition, there was no significant difference in fatigue severity at T0 (mean (SD): dropouts: 43.54 (14.42); completers: 44.25 (15.33); t_(675)_: -0.53, *P* = 0.593).

### Questionnaires

Level of fatigue over the past 2 weeks was assessed with the Dutch 20-item Checklist Individual Strength (CIS-20R) [25]. The questionnaire consists of four subscales: severity of fatigue, concentration, motivation, and physical activity. The total fatigue score is the sum score of all items (range, 20 to 140). The questionnaire is well validated and has good reliability.

Collected demographics and deployment characteristics included age and rank during deployment, gender, educational level, number of previous deployments, and use of medication (non-systemic glucocorticoids (nasal spray or crème), antihistamines, cholesterol-lowering medications, and anti-hypertensives). Exposure to deployment-stressors was assessed with a 13-item checklist during the T1 assessment (available as supplementary material in 22).

### IL-1β-reactivity

Whole blood, diluted 1:10 with RPMI-1640 (Gibco, Grand Island, NY, USA), was stimulated with human interleukin (IL)-1β (Pepro Tech Inc, Rocky Hill, NJ, USA) for 24 h at 37 °C/5% CO2 in 96-well flat-bottomed plates. The final concentrations of IL-1β were: 0, 1, 3, 10, 30 ng/mL. IL-1β doses in this range are frequently used in *in vitro* experiments in various tissues [26-29]. Supernatants were stored at −80 °C. In a pilot analysis, the level of IL-6, TNF-a, IL10, and IL-8 were determined by ELISA (Sanquin, the Netherlands). IL-6, TNF-a, IL-10, and IL-8 were selected as initial read-outs, because they represent characteristic cytokines of respectively the pro-inflammatory, anti-inflammatory, and chemoattractive cytokine spectrum. In an initial screening of samples from 37 individuals it became apparent that IL-1β did not induce IL-10 production. In addition, screening approximately 750 random samples revealed that in response to the lowest dose of IL-1β, the TNF-α level was below the detection limit in 51% of the samples and IL-6 was not detectable in 11.5% of the samples. Moreover, after stimulation with 30 ng/mL IL-1β, we did not detect TNF-a in 15% and IL-6 in 2.5% of the samples. In contrast, IL-8 appeared to be robustly induced by IL-1β, with only 5.4% of the values below the detection limit after stimulation with 1 ng/mL IL-1β and 0.5% below the detection limit after stimulation with 30 ng/mL IL-1β. Therefore, we selected IL-8 as a read-out. Further analyses showed that there was a robust, dose-dependent increase in IL-8 in response to stimulation by IL-1β.

Absolute numbers of monocytes, granulocytes, lymphocytes, and CD3+ T-cells were calculated from a total leukocyte count. To determine the response to LPS, whole blood was diluted 1:10 with RPMI-1640 (Gibco, Grand Island, NY, USA), and stimulated with lipopolysaccharide (LPS, Escherichia Coli 0127:B8, Sigma, final concentrations 1 ng/mL) for 24 h at 37 °C/5% CO2 in 96-well flat-bottomed plates. Supernatants were stored at -80C and IL-8 concentrations were determined using a multiplex cytokine assay [30]. We used a dose of 1 ng/mL LPS, since preliminary analysis of a dose–response curve (0, 0.01, 0.1, and 1 ng/mL LPS) revealed that a plateau in IL-8 production was reached at a dose of 1 ng/mL LPS.

### Statistics

Statistical analyses were conducted using PASW/SPSS 18.0. Differences between groups were considered significant at *P* < 0.05. All continuous variables were tested for normality and log-transformed when necessary. A limited number of missing values were present due to technical and handling problems (<7.5% for each variable). Outliers were removed if z-values were outside the range of ± 3.29 [31] (<2% for each variable).

Differences between groups in continuous demographic and deployment characteristics were assessed with t-tests. Differences in non-continuous demographic variables between groups were tested with Chi-square (*χ*^2^) tests. Repeated measures ANOVA was used to analyze the dose–response of IL-8 production after stimulation with increasing doses of IL-1β at T2. In addition, repeated measures ANOVA was used to analyze the longitudinal course of CIS-20R total scores, IL-1β-induced IL-8 production, non-stimulated IL-8 production, cell subsets, and LPS-induced IL-8 production. Time was used as within-subjects factor and group as between-subjects factor. A Greenhouse-Geisser correction was applied when sphericity was violated and ε ≤ 0.75. A Huyn-Feldt correction was applied if sphericity was violated and ε > 0.75 [31]. *Post hoc* t-tests with Bonferroni correction were used for follow-up of significant effects. In addition, significant group x time interactions differences were followed by simple effects analyses [31]. Pearson’s r correlations were used to investigate associations between IL-1β-induced IL-8 production and demographic and deployment characteristics for each assessment point. Demographic and deployment characteristics that significantly correlated with IL-8 production were included as covariates in the repeated measures ANOVA. Non-transformed data are presented in all tables and figures.

## Results

### Participant characteristics and longitudinal course of fatigue symptoms

Our aim was to investigate whether participants with and without severe fatigue after return from military deployment differed in IL-1β reactivity of peripheral blood cells *in vitro*. For that purpose, we decided to use a dichotomous approach in which participants were divided into groups with and without severe fatigue (that is, a score above or below the cutoff of 81 on the CIS-20R total score) at 6 months after return (T2).

**Figure 1:**
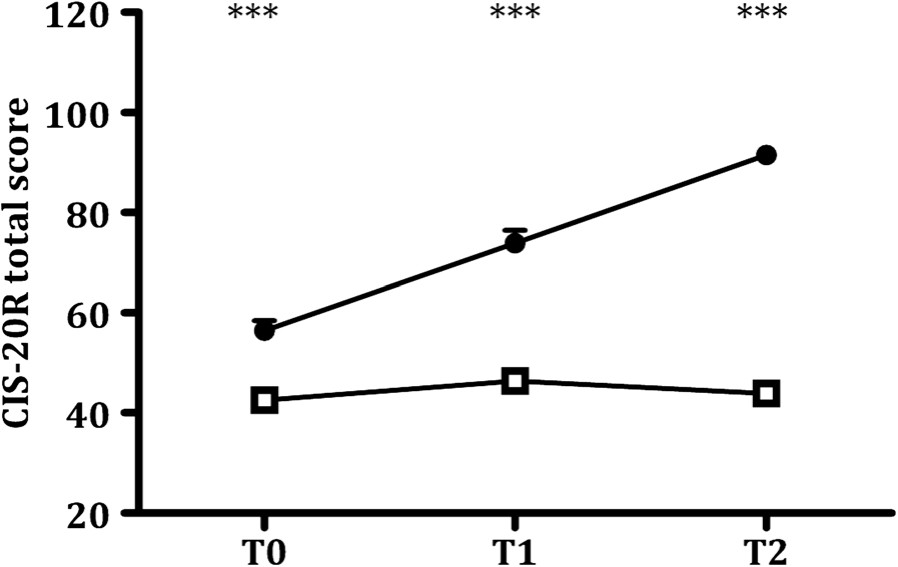
**Dose–response curves for IL-1β-induced IL-8 production 6 months post-deployment for the fatigued and non-fatigued group.** Whole blood samples obtained 6 months after deployment (T2) from participants assigned to the fatigued group (black circles, *n* = 62) and non-fatigued group (white squares, *n* = 401) were stimulated for 24 h with increasing concentrations of IL-1β. The amount of IL-8 in the culture supernatant was measured by ELISA. IL-1β induced a dose-dependent increase in IL-8 production in both groups (dose: F_(2.00, 924.02)_: 602.82, *P* < 0.001), but the dose–response curve differed between the two groups (group: F_(1,461)_: 4.55, *P* < 0.01; dose x group: F_(2.00, 924.02)_: 7.94, *P* < 0.001). After applying Bonferroni correction, the fatigued group had significantly higher IL-8 production than the non-fatigued group after administration of 10 ng/mL (*P* < 0.01) and 30 ng/mL IL1β (*P* < 0.01). Data are presented as mean ± SEM. #*P* < 0.05, significant before Bonferroni correction, but not after Bonferroni correction, **P < 0.01, significant after Bonferroni correction.

We first analyzed the difference in the longitudinal course of symptoms (Figure 1). As expected, we observed a significant difference in the longitudinal course of fatigue symptoms between the fatigued and non-fatigued group (time: F_(1.98, 970.51)_: 138.17, *P* < 0.001; group: F_(1,491)_: 283.73, *P* < 0.001; interaction effect time x group: F_(1.98, 970.51)_: 118.40, *P* < 0.001). To further interpret this result, we analyzed the longitudinal course of symptom development for both groups separately. The participants with severe fatigue at T2 showed a strong increase in fatigue severity after deployment compared to fatigue levels at T0. Moreover, the fatigue severity at T2 had continued to increase compared one month after deployment (T1) (time: F_(2, 128)_: 89.844, *P* < 0.001; change from T0-T1: *P* < 0.001, T0-T2: *P* < 0.001, T1-T2: *P* < 0.001). The non-fatigued group had slightly increased fatigue questionnaire scores at T1 compared to T0. However, their questionnaire scores had returned to baseline level at T2 (time: F_(1.97, 843.02)_: 13.123, *P* < 0.001; change from T0-T1: *P* < 0.001, T0-T2: *P* = 0.163, T1-T2: *P* < 0.05).

Interestingly, although participants with severe fatigue before deployment (T0) were excluded from the analyses, participants with severe fatigue at T2 had higher fatigue questionnaire scores than the non-fatigued group at all assessment points (all time-points: *P* < 0.001).

We did not observe any significant group differences in demographic and deployment characteristics between the fatigued and non-fatigued participants (Table 1).

**Table 1:** Characteristics of the fatigued and non-fatigued group

	**Fatigued group (*n*** **= 65)**	**Non-fatigued group (*n*** **= 439)**	***P*value**
Age during deployment	29.29 (9.19)	28.47 (8.96)	0.490
Previous deployments (*n*)	1.05 (1.31)	0.89 (1.20)	0.320
Deployment stressors (*n*)	5.53 (2.46)	4.98 (2.92)	0.106
Gender			0.667
Male	60 (92.3%)	398 (90.7%)	
Female	5 (7.7%)	41 (9.3%)	
Rank			0.454
Soldiers	20 (30.8%)	179 (40.8%)	
Corporals	13 (20.0%)	84 (19.1%)	
Non-commissioned officers	20 (30.8%)	112 (25.2%)	
Officers	12 (18.5%)	64 (14.6%)	
Education			0.744
Low	23 (35.9%)	170 (39.3%)	
Middle	32 (50.0%)	215 (49.7%)	
Higher	9 (14.1%)	48 (11.1%)	

Data represent mean (standard deviation) or frequency (percentage).

### IL-1β-induced IL-8 production 6 months after deployment

**Figure 2:**
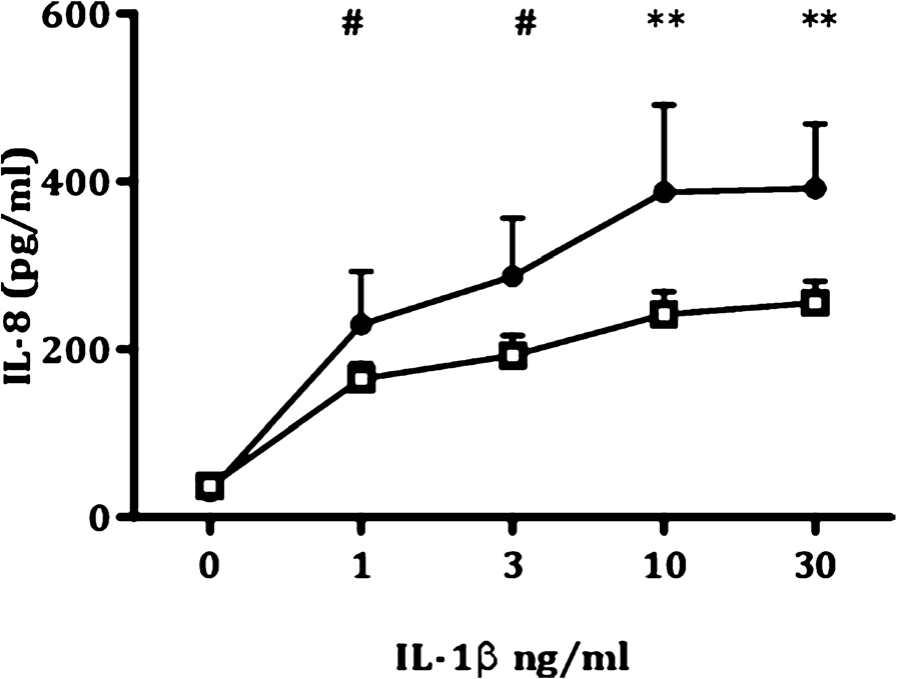
**Longitudinal course of fatigue questionnaire scores for the fatigued and non-fatigued group.** Longitudinal course of CIS-20R total scores for the severely fatigued group (black circles, *n* = 65) and non-fatigued group (white squares, *n* = 428). Fatigue severity was assessed before deployment (T0) and 1 month (T1) and 6 months (T2) after return from deployment. The longitudinal course of CIS-20R scores differed between the two groups (time: F_(1.98, 970.51)_: 138.17, *P* < 0.001; group: F_(1,491)_: 283.73, *P* < 0.001; interaction effect time x group: F_(1.98, 970.51)_: 118.40, *P* < 0.001). However, participants with severe fatigue at T2 had higher fatigue questionnaire scores than the non-fatigued group at all assessment points (T0: *P* < 0.001, T1: *P* < 0.001, T2: *P* < 0.001). Data are presented as mean ± SEM. ***P < 0.001.

We first investigated whether the fatigued and non-fatigued group differed in IL-1β-induced IL-8 production 6 months after return from deployment. For this purpose, we analyzed group differences in the dose–response curve for IL-1β-induced IL-8 production in cultures of whole blood collected at the assessment 6 months after deployment (T2). Repeated measures ANOVA showed that IL-1β induced a dose-dependent increase in IL-8 production in both groups (dose: F_(2.00, 924.02)_: 602.82, *P* < 0.001). Interestingly, the dose–response curve of IL-1β-induced IL-8 production differed between the groups (group: F_(1,461)_: 4.55, *P* < 0.01; dose x group: F_(2.00, 924.02)_: 7.94, *P* < 0.001) (Figure 2). *Post hoc* tests for group differences in IL-8 production for each dose of IL-1β revealed that the fatigued group had significantly higher IL-8 production than the non-fatigued group after administration of 1 ng/mL (*P* < 0.05), 3 ng/mL (*P* < 0.05), 10 ng/mL (*P* < 0.01), and 30 ng/mL IL1β (*P* < 0.01). The group difference at 10 ng/mL and 30 ng/mL IL-1β remained significant after applying a Bonferroni correction (significant *P* value $\left(\alpha \right)=0.05/5=.01)$.

The observed difference in the dose–response curve of IL-1β-induced IL-8 production between the fatigued and non-fatigued group was not paralleled by significant group differences in the number of monocytes 6 months after deployment (t_(470)_: 0.30, *P* = 0.767), granulocytes (t_(468)_: -1.48, *P* = 0.140), lymphocytes (t_(468)_: -0.65, *P* = 0.514), or CD3+ T-cells (t_(462)_: -0.64, *P* = 0.525) at T2.

### Longitudinal course of IL-1β-induced IL-8 production

We investigated whether the higher IL-1β -induced IL-8 production in the fatigued group was already present prior to deployment or whether the observed group difference developed over time. For that purpose, we compared the longitudinal course of IL-1β-induced IL-8 production between the fatigued and non-fatigued group.

At T2, we observed the largest group difference in IL-1β-induced IL-8 production between the fatigued and non-fatigued group after stimulation with 30 ng/mL IL-1β. Therefore, we selected this dose for these subsequent analyses.

**Figure 3:**
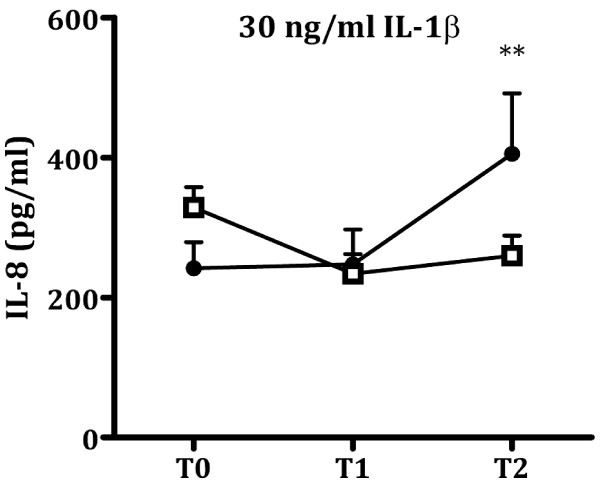
**Longitudinal course of IL-1β-induced IL8-production for the fatigued and non-fatigued group.** Whole blood samples obtained from participants assigned to the fatigued group (black circles, *n* = 55) and non-fatigued group (white squares, *n* = 353) were stimulated for 24 h with 30 ng/mL IL-1β. The amount of IL-8 in the culture supernatant was measured by ELISA, at the assessments before deployment (T0), and 1 month (T1) and 6 months (T2) after return from deployment. The longitudinal course of IL-1β-induced IL-8 production differed between the two groups (time: F_(1.96; 794.68)_: 2.60, *P* = 0.076; group: F_(1,406)_: 5.27, *P* < 0.05; time x group: F_(1.96: 794.68)_: 4.30, *P* < 0.05). The IL-1β-induced IL-8 production of the non-fatigued group decreased over time (time: F_(1.96, 690.66)_: 13.57, *P* < 0.001). In contrast, the amount of IL-1β-induced IL-8 production of the fatigued group did not significantly change over time (time: F_(1.80, 97.19)_: 2.30, *P* = 0.111). IL-1β-induced IL-8 production significantly differed between the fatigued and non-fatigued group at T2 (*P* < 0.01), but not at T0 (*P* = 0.867) or T1 (*P* = 0.062). Data are presented as mean ± SEM. **P < 0.01.

We observed a significant difference in the longitudinal course of IL-1β-induced IL-8 production between the fatigued and non-fatigued group (Figure 3; time: F_(1.96; 794.68)_: 2.60, *P* = 0.076; group: F_(1,406)_: 5.27, *P* < 0.05; time x group: F_(1.96, 794.68)_: 4.30, *P* < 0.05). To further interpret this result, we analyzed the longitudinal course of IL-1β-induced IL-8 production for both groups separately. IL-1β-induced IL-8 production of the non-fatigued group decreased after deployment (time: F_(1.96, 690.66)_: 13.57, *P* < 0.001). Both at T1 and T2, IL-1β-induced IL-8 production in the non-fatigued group was significantly lower than at T0 (change from T0-T1: *P* < 0.001; T0-T2: *P* < 0.001; T1-T2: *P* = 1.000). In contrast, the amount of IL-1β-induced IL-8 production of the fatigued group did not significantly change over time, and if anything tended to increase (time: F_(1.80, 97.19)_: 2.30, *P* = 0.111).

We also analyzed whether the actual amount of IL-1β-induced IL-8 production differed between the fatigued and non-fatigued group at the three time points tested. *Post hoc* t-tests with Bonferroni correction (significant *P* value (α) = .05/3 = 0.016) revealed that the IL-8 production of the fatigued and non-fatigued group did not differ at T0 (*P* = 0.867) and T1 (*P* = 0.062). As already described, the IL-8 production of the fatigued and non-fatigued group did significantly differ at T2 (*P* < 0.01).

The observed difference in the longitudinal course of IL-1β-induced IL-8 production between the fatigued and non-fatigued group was not paralleled by significant group differences in the longitudinal course of the number of monocytes, granulocytes, lymphocytes, or CD3+ T-cells over time (Table 2).

**Table 2:** Differences in the longitudinal course of various cell subsets between the fatigued and non-fatigued group

	**Time effect**	**Group effect**	**Group x time effect**
Monocytes	F = 16.91, *P* < 0.001	F = 0.83, *P* = 0.362	F = 0.94, *P* = 0.388
Granulocytes	F = 5.88, *P* < 0.05	F = 0.23, *P* = 0.635	F = 2.67, *P* = 0.070
Lymphocytes	F = 5.93, *P* < 0.05	F = 2.37, *P* = 0.095	F = 2.15, *P* = 0.144
T-cells (CD3+)	F = 2.74, *P* = 0.067	F = 2.03, *P* = 0.133	F = 0.46, *P* = 0.497

### Longitudinal course of LPS-induced IL-8 production

**Figure 4:**
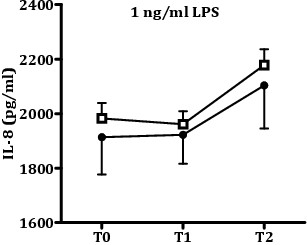
**Longitudinal course of LPS-induced IL8-production for the fatigued and non-fatigued group.** Whole blood samples obtained from participants assigned to the fatigued group (black circles, *n* = 53) and non-fatigued group (white squares, *n* = 388) were stimulated for 24 h with 1 ng/mL LPS and IL-8 levels in the culture supernatant were quantified. Samples were collected before deployment (T0), and 1 month (T1) and 6 months (T2) after return from deployment. The amount of LPS-induced IL-8 production did not change over time and there were no significant differences between the fatigued and non-fatigued group (time: F_(1.90, 934.51)_: 1.87, *P* = 0.157; group: F_(1,439)_: 0.36, *P* = 0.551; time x group: F_(1.90, 934.51)_: 0.10, *P* = 0.900). Data are presented as mean ± SEM.

Next, we addressed the question whether the observed difference in the longitudinal course of IL-8 production between fatigued and non-fatigued individuals is specific for IL-1β-signaling or represents a general group difference in the capacity to produce IL-8. To that end, we compared the longitudinal course of LPS-induced IL-8 production between the fatigued and non-fatigued group. The data presented in Figure 4 demonstrates that LPS-induced IL-8 production did not change over time (F_(1.90, 934.51)_: 1.87, *P* = 0.157). Moreover, there were no significant differences between the fatigued and non-fatigued group (group: F_(1,439)_: 0.36, *P* = 0.551; interaction group x time: F_(1.90, 934.51)_: 0.01, *P* = 0.900).

### Influence of demographic and deployment characteristics

To ascertain that our results were not influenced by confounding factors, we examined correlations between demographic and deployment characteristics and IL-1β-induced IL-8 production for each assessment point separately (Table 3). Demographic and deployment characteristics that correlated significantly with IL-8 production on at least one assessment point were subsequently included as covariates in our analyses. After inclusion of age, rank, educational level, and the number of reported deployment stressors in the analysis, the longitudinal course of Il-1β induced IL-8 production remained significantly different between fatigued and non-fatigued individuals (time: F_(1.98, 745.47)_: 9.60, *P* < 0.001; time x group interaction: F_(1.94, 745.47)_: 3.89, *P* < 0.05; group: F_(1,376)_: 5.65, *P* < 0.05).

**Table 3:** Pearson’s correlations between IL-8 production after stimulation with IL-1β and demographic and deployment characteristics

	**IL-8 at T0**	**IL-8 at T1**	**IL-8 at T2**
Age	−0.132^a^	−0.045	−0.208^b^
Gender	0.078	−0.064	0.021
Previous deployments (*n*)	−0.035	−0.016	−0.036
Rank	−0.167^b^	−0.002	−0.160^c^
Educational level	−0.123^c^	0.021	−0.080
Medication use (y/n)	0.001	0.026	0.001
Deployment stressors (*n*)		0.149^c^	0.063
Injury during deployment (y/n)		−0.065	0.032

IL-8 production after stimulation with 30 ng/mL IL-1β was assessed before deployment (T0) and 1 month (T1) and 6 months (T2) after return from deployment.

^a^*P* < 0.05.

^b^*P* < 0.001.

^c^*P* < 0.01.

## Discussion

This study was designed based on the concept that the response of the body to a regulatory mediator is not only determined by the concentration of the mediator, but also by the reactivity of the target cells to regulation by a particular mediator [14]. Our findings indicate that assessment of the reactivity of immune cells to IL-1β *in vitro* may represent a promising novel approach to investigate the relation between severe fatigue and pro-inflammatory cytokine signaling. Fatigue and IL-1β-induced IL-8 production by peripheral blood cells *in vitro* were assessed in a unique longitudinal prospective design within a large cohort of soldiers (*n* = 504) measured before, and at two time-points after deployment to a combat zone in Afghanistan. None of the included participants reported severe fatigue prior to the deployment, and therefore the observed effects are most likely associated with the development of severe fatigue in response to the deployment.

At 6 months after return from military deployment, the participants with severe fatigue had higher IL-1β-induced IL-8 production than the non-fatigued participants, indicating that the peripheral blood cells of fatigued participants had a higher reactivity to IL-1β than those of the non-fatigued group. The observed group-difference in IL-1β-induced IL-8 production was specifically associated with a group difference in the reactivity of peripheral blood cells to stimulation with IL-1β, because we did not observe a group difference in LPS-induced IL-8 production. In addition, the increased IL-1β-induced IL-8 production in the fatigued group could not be attributed to group differences in the cellular composition of the peripheral blood.

Investigation of the longitudinal course of IL-1β-induced IL-8 production revealed that the group difference in IL-1β reactivity between participants with and without severe fatigue after return from deployment was not a pre-existing characteristic, but had developed over time. The IL-1β-induced IL-8 production of non-fatigued participants had decreased 1 and 6 months after deployment compared to the assessment before deployment. This finding indicates that the leukocytes of non-fatigued participants had become less reactive to stimulation with IL-1β over time. In the group of participants with severe fatigue 6 months after deployment, we did not observe this decrease in IL-1β-induced IL-8 production over time.

During deployment to Afghanistan the participants in this study encountered a variety of stressors, such as armed combat, improvised explosive devices (IEDs), mortar attacks, and witnessing colleagues or civilians being injured or killed as a result. Given the severity of these deployment stressors, we interpret the 4-month deployment as prolonged stress. We did not include a non-deployed control group and therefore, we cannot conclude that the observed changes in fatigue and IL-1β-induced cytokine production result from the stress of the deployment. However, it is unlikely that the observed effects can be attributed to aspecific time-effects such as the year or season of assessment, since we included military personnel in several subsequent cohorts between 2006 and 2009.

It has been reported previously that severe or chronic stress, such as expected to occur during deployment, results in increased levels of circulating pro-inflammatory cytokines [32], up-regulated expression of genes with NFκB response elements and down-regulated expression of genes with GR response elements in leukocytes [33,34]. In the current study, the participants who did not develop fatigue 6 months after deployment showed a decrease in their reactivity to IL-1β *in vitro* after return from deployment. Interestingly, in a previous study the up-regulation of gene expression with NFκB response elements in chronically stressed individuals was paralleled by increased serum IL-1RA, which could decrease IL-1β capacity [34]. These data indicate that in periods of severe or chronic stress, adaptive mechanisms may develop to reduce IL-1 reactivity. Our finding that the IL-1β-induced IL-8 production in the participants with severe fatigue after deployment did not decrease over time could indicate that these participants have adapted less well to the stress experienced during the deployment.

At present, the underlying mechanism for the observed higher IL-1β-induced IL-8 production in participants with severe fatigue after deployment as compared to the non-fatigued group remains unknown. It is known that activation of the transcription factor NFκB, in combination met NF-IL6, is essential and sufficient to induce up-regulation of IL-8 expression after stimulation with IL-1β [35]. LPS-induced Il-8 production is also dependent on activation of NFκB, but in this case in combination with AP-1 [35]. Thus, IL-1β and LPS both induce IL-8 via transcription factor NFκB, but in addition use separate other transcription factors. Therefore it is possible that the group difference in IL-1β-induced IL-8 production and not LPS-induced IL-8 production is mediated by a preferential activation of NF-IL6 in the fatigued group after stimulation with IL-1β. It is also possible that the mechanism(s) involved in the development of the group difference in IL-1β reactivity is located upstream of transcription factor activation, that is, at the level of IL-1 receptor expression and/or signaling. The type I IL-1 receptor (IL-1RI) mediates the biological effects of IL-1α and IL-1β [18]. The type II IL-1 receptor (IL-1RII) binds IL-1α and IL-1β with high affinity, but does not signal: it functions as a ‘decoy’ receptor, which prevents signal transduction via IL-1RI and thereby negatively regulates IL-1 signaling [18]. The higher response to IL-1β in the fatigued group compared to the non-fatigued group may hypothetically have resulted from higher IL-RI levels, lower IL-1RII levels, or higher IL-1RI signaling to downstream targets such as the transcription factors mentioned above.

In addition, it is possible that IL-1 receptor antagonist (IL-1RA) contributes to the observed group difference in IL-1β reactivity 6 months after deployment. IL-1RA can negatively regulate IL-1β signaling, since IL-1RA binding to IL-1RI does not elicit signal transduction, but inhibits activation of the receptor by IL-1β [36]. It is possible that the fatigued group had lower levels of circulating IL-1RA than the non-fatigued group at 6 months after deployment. Miller *et al*. [34] observed that mean serum IL-1RA levels in individuals with chronic caregiver stress were 450 pg/mL, while mean serum IL-1RA levels of non-stressed healthy controls were 200 pg/mL. On the basis of these data, we expect that serum IL-1RA levels in our participants are likely to be in the 200 to 450 pg/mL range. The final concentration of IL-1RA in our whole blood culture system (final whole blood dilution = 1:20) is therefore expected to be 10 to 22.5 pg/mL. However, a 10- to 100-fold excess of IL-1RA is necessary to block the binding of IL-1β to the IL-1R [37]. Therefore, the expected IL-1RA levels in our *in vitro* cultures are probably too low to block the effects of the dose of IL-1β we used. In addition, if group differences in the level of the competitive inhibitor IL-1RA were responsible for the observed group difference in IL-1β-induced IL-8 production, the largest group differences would be expected at the lower doses of IL-1β, instead of at the highest doses of IL-1β.

We observed that the peripheral blood cells of the fatigued participants reacted differently to stimulation with a pro-inflammatory cytokine, that is, IL-1β, *in vitro*. It remains to be determined whether the observed group difference in reactivity to IL-1β *in vitro* is also present *in vivo*. In rodents it has been shown that cytokine levels in the brain are the mirror image of cytokine levels in the periphery [15]. For example, peritoneal administration of IL-1β in rats up-regulate mRNA expression of various pro-inflammatory cytokines in the brain [38]. Future research should investigate whether differences in brain responses to cytokines contribute to the development of fatigue.

A limitation of the current study is that we did not formally investigate the presence of medical conditions that may have influenced the IL-1β-sensitivity of peripheral blood cells or the experienced levels of fatigue. However, participants were physically fit for military deployment and therefore the presence of major medical conditions prior to deployment is highly unlikely. In addition, the presence of injuries after deployment and medication use during the three assessments was very limited. Moreover, medication use and sustained injuries did not significantly correlate with IL-1β-induced IL-8 production. It thus seems highly unlikely that the presence of medical conditions influenced our results.

We are the first to report that the response of peripheral blood cells to IL-1β *in vitro* differs between soldiers with and without severe fatigue 6 months after return from deployment. Six months after return from deployment, the group who had become severely fatigued had higher IL-1β-induced IL-8 production than the non-fatigued group. When analyzing the longitudinal course of IL-1β reactivity, we observed that this group difference had developed in response to the deployment, since only in the non-fatigued group the IL-1β-induced IL-8 production had decreased after deployment. These findings indicate that investigating the reactivity of the immune system to stimulation with IL-1β is a promising novel method to study the association between behavioral abnormalities and pro-inflammatory cytokine signaling.

## Competing interests

All authors declare that they have no competing interests.

## Authors’ contributions

MvZ, AK, EG, and CH designed the current study. EV, AK, and CH designed the larger longitudinal study and wrote the study protocol. Literature searches were performed by MvZ, AK, EG, and CH. AK and MM handled the logistics concerning all collected samples and performed the assays. MvZ performed the statistical analyses and wrote the first draft of the manuscript. All authors contributed to and have approved the final manuscript.
